# Bicarbonate‐sensing soluble adenylyl cyclase is present in the cell cytoplasm and nucleus of multiple shark tissues

**DOI:** 10.14814/phy2.13090

**Published:** 2017-01-20

**Authors:** Jinae N. Roa, Martin Tresguerres

**Affiliations:** ^1^ Marine Biology Research DivisionScripps Institution of OceanographyUniversity of California San Diego9500 Gilman DriveLa JollaCalifornia92093USA

**Keywords:** adcy10, cAMP, cyclic AMP, microdomain, pH sensing, phosphorylation, regulation of gene expression, signal transduction

## Abstract

The enzyme soluble adenylyl cyclase (sAC) is directly stimulated by bicarbonate (HCO_3_^−^) to produce the signaling molecule cyclic adenosine monophosphate (cAMP). Because sAC and sAC‐related enzymes are found throughout phyla from cyanobacteria to mammals and they regulate cell physiology in response to internal and external changes in pH, CO_2_, and HCO_3_^−^, sAC is deemed an evolutionarily conserved acid‐base sensor. Previously, sAC has been reported in dogfish shark and round ray gill cells, where they sense and counteract blood alkalosis by regulating the activity of V‐type H^+^‐ ATPase. Here, we report the presence of sAC protein in gill, rectal gland, cornea, intestine, white muscle, and heart of leopard shark *Triakis semifasciata*. Co‐expression of sAC with transmembrane adenylyl cyclases supports the presence of cAMP signaling microdomains. Furthermore, immunohistochemistry on tissue sections, and western blots and cAMP‐activity assays on nucleus‐enriched fractions demonstrate the presence of sAC protein in and around nuclei. These results suggest that sAC modulates multiple physiological processes in shark cells, including nuclear functions.

## Introduction

The acid‐base status of physiological fluids is largely dictated by pH, CO_2_, and bicarbonate (HCO_3_^−^) levels, and acid‐base status is one of the most tightly regulated physiological processes because even small deviations from the acid‐base set point can affect the structure and function of enzymes and other cellular components. In animals, the acid‐base status of extracellular fluids is regulated by specialized organs such as gills, lungs, and kidneys, which provide a stable acid‐base environment to the rest of the cells in the body.

An essential requirement for acid‐base regulation is the ability to sense acid‐base conditions in the first place. One evolutionarily conserved acid‐base sensing mechanism relies on the enzyme soluble adenylyl cyclase (sAC, adcy10), which is directly regulated by HCO_3_^−^ to produce the ubiquitous messenger molecule cyclic adenosine monophosphate (cAMP) (Buck et al. 1999; Chen et al. 2000). In vivo, the HCO_3_^−^ that stimulates sAC can originate from the carbonic anhydrase‐catalyzed hydration of CO_2_ from external or metabolic origin, from intracellular CO_2_/HCO_3_^−^ elevations resulting from changes in external pH, or from HCO_3_^−^ entry through ion transporting proteins (reviewed in Tresguerres et al. 2014, 2011; Tresguerres 2014). In recent years, sAC has been established as a sensor and regulator of blood acid‐base status in the mammalian kidney (Paunescu et al. 2008, 2010; Gong et al. 2010) and in gills of dogfish shark (Tresguerres et al. 2010c) and round rays (Roa and Tresguerres 2016), as well as in neuronal chemo‐sensitive cells known to regulate lung ventilation (Imber et al. 2014; Nunes et al. 2014). In addition to sAC, the organs mentioned above posses other putative acid‐base sensors (reviewed in Tresguerres et al. 2010a), some of which also produce cAMP. For example, some cells in the mammalian kidney express H^+^‐sensing G‐protein coupled receptors (GPCRs) linked to transmembrane adenylyl cyclases (tmACs) (Ludwig et al. 2003; Sun et al. 2010, 2015), and round ray gill acid‐ and base‐secreting cells contain both sAC and tmACs (Roa and Tresguerres 2016). The existence of multiple sources of cAMP raises questions about how downstream signaling is regulated and coordinated. Simply put, how can a single messenger molecule regulate multiple, often antagonistic, processes in a same cell? In response to these questions, the “cAMP signaling microdomain” model has been proposed, whereby cAMP‐producing enzymes are tethered to the cell membrane, to cytoskeletal components, and inside the nucleus and mitochondria, together with phosphodiesterase enzymes that degrade cAMP thus preventing cAMP diffusion and cross‐communication between cAMP‐producing focal points (reviewed in Cooper 2003; Lefkimmiatis and Zaccolo 2014; Tresguerres et al. 2011). Although this model is supported by studies in several mammalian cells such as HT22, AtT20, and 3T3‐L1 from brain, pituitary, and embryonic tissues, respectively (Inda et al. 2016), evidence in other animals is mostly lacking. Thus, the first aim of this study was to explore whether acid‐ and base‐secreting gill cells from the leopard shark (*Triakis semifasciata*) have the two sources of cAMP (sAC and tmACs), and therefore if the cAMP microdomain model is widespread in vertebrate animals.

In addition to acid‐base regulatory epithelia, variations in pH, CO_2_, and HCO_3_^−^ levels are known to affect virtually every physiological process. While many of these effects may be due to the direct action of H^+^ or HCO_3_^−^ on specific effector proteins (Sadowski et al. 1976; Aronson et al. 1982; Srivastava et al. 2007), many other effects have been recently been found to be mediated by sAC‐derived cAMP regulation. For example, in response to extracellular alkalosis, sAC has been shown to simulate ion and water transport across fish intestinal epithelia (Tresguerres et al. 2010b; Carvalho et al. 2012), and mammalian renal collecting duct (Hallows et al. 2009) and pancreas (Strazzabosco et al. 2009), heart beat rate in hagfish recovering from anoxia (Wilson et al. 2016), sperm flagellar movement and capacitation (Esposito et al. 2004; Hess et al. 2005), and metabolic coupling between neurons and astrocytes in rat (Choi et al. 2012), among others Tresguerres et al. 2014 Tresguerres et al. 2010a. Because sharks experience a pronounced blood alkalosis in the postfeeding period, also known as the “alkaline tide” (Wood et al. 2005, 2007a, 2010), HCO_3_^−^‐stimulated sAC is a good candidate to mediate multiple physiological adjustments. Thus, the second objective of this study was to examine the presence of sAC in shark organs that accomplish physiological functions known to be modulated by acid‐base conditions, and for which there is evidence for the involvement of sAC in similar organs from mammals and fish. With this in mind, we examined the rectal gland, cornea, muscle, heart, and intestine.

Finally, sAC has been reported in the nucleus of some mammalian cells, where it is proposed to form a cAMP signaling microdomain that regulates gene expression by cAMP/PKA‐dependent phosphorylation of transcription factors (Zippin et al. 2003, 2004). Metabolic alkalosis and the postfeeding alkaline tide are known to upregulate the abundance of various proteins in gill (Tresguerres et al. 2006) and rectal gland (Dowd et al. 2008), as well as the activity of metabolic and iono‐regulatory enzymes in rectal gland (Walsh et al. 2006; Wood et al. 2008). Thus, the third and final aim of this study was to determine whether sAC is present in the nucleus of shark gill and rectal gland cells, as a first step to identifying the molecular mechanisms that may modulate gene transcription in response to alkalosis.

## Methods

### Experimental animals

All experiments were approved by the SIO‐UCSD animal care committee under protocol number #S10320 in compliance with the IACUC guidelines for the care and use of experimental animals. Leopard sharks were caught from La Jolla Shores, CA, housed in tanks with flowing seawater, and were fed chopped squid or mackerel three times a week.

### Tissue sampling

Specimens were killed by an overdose of tricaine methanesulfonate (0.5 g·L^−1^) and samples from the various organs were taken for the different experimental procedures. For immunohistochemistry, samples were fixed in 0.2 mol/L cacodylate buffer, 3.2% paraformaldehyde, 0.3% glutaraldehyde for 6 h, transferred to 50% ethanol for 6 h, and stored in 70% ethanol until further processing as described in Tresguerres et al. (2005). For western blot analysis, gill samples were flash frozen in liquid nitrogen and stored at −80°C. For nuclei isolation, gill and rectal gland samples were placed ice‐cold shark saline (280 mmol/L NaCl, 6 mmol/L KCl, 5 mmol/L NaHCO_3_, 3 mmol/L MgCl_2_, 0.5 mmol/L NaSO_4_, 1 mmol/L Na_2_HPO_4_, 350 mmol/L urea, 70 mmol/L trimethylamine N‐oxide, 5 mmol/L glucose, 1:100 protease inhibitor cocktail [1 mmol/L dithiothreitol, 1 mmol/L phenylmethylsulfonyl fluoride, 10 mmol/L benzamidine hydrochloride hydrate; SigmaAldrich], pH 7.7) and processed immediately.

### Nuclei isolation

Nuclei were isolated from the gills and rectal gland of leopard sharks using methods adapted from research on nuclear sAC in mammals (Zippin et al. 2004).A quantity of 100 mg of fresh tissues were homogenized (30 sec, 4°C) by hand using a glass pestle tissue grinder with 1 mL of shark saline and differential centrifugation used to separate nuclear, membrane, and cytoplasmic fractions. Initially, homogenates were centrifuged for 2 min at 1000*g*. Cytoplasmic and membrane fractions remained in the supernatant and were removed, while the nucleus‐containing pellet was resuspended in a mix of fresh shark saline, a sucrose solution (1 mol/L), and Optiprep density gradient medium (1:1:2) (SigmaAldrich, St. Louis, MO) and then centrifuged for 20 min at 10,000*g* to obtain a nuclear‐enriched pellet.

### Antibodies and reagents

Two custom‐made polyclonal rabbit antibodies were used: one specifically recognizes an epitope in the second catalytic domain of dogfish sAC (dfsAC) (INNEFRNYQGRINKC) (Tresguerres et al. 2010c) and the other specifically recognizes a conserved region in the VHA B‐subunit (AREEVPGRRGFPGY) (Roa et al. 2014). Both antibodies were custom made and produced GenScript (Piscataway, NJ). Na^+^/K^+^‐ATPase (NKA) was immunolocalized using a monoclonal mouse antibody raised against the chicken *α*‐subunit (Lebovitz et al. 1989), purchased from the Developmental Studies Hybridoma Bank (Iowa City, IA). Horseradish peroxidase (HRP)‐conjugated goat anti‐mouse and goat anti‐rabbit antibodies (BioRad, Hercules, CA) were used for immunoblot detection. Fluorescent goat anti‐mouse Alexa Fluor 568 and goat anti‐rabbit Alexa Fluor 488 and Alexa Fluor 555 secondary antibodies (Invitrogen, Grand Island, NY) were used for immunolocalization. BODIPY‐FL Forskolin dye (ThermoFisher Scientific, Waltham, MA) was used to determine tmAC localization; the BODIPY‐FL Forskolin conjugate utilizes forskolin, a compound that selectively binds to tmACs (Seamon and Daly 1981; Buck et al. 1999), to localize tmACs and has recently been used in mammalian cell cultures (Calebiro et al. 2009; Kuna et al. 2013), zebrafish (Kumai et al. 2014), and round rays (Roa and Tresguerres 2016). KH7 was a kind gift of Dr. Lonny Levin and Dr. Jochen Buck (Weill Cornell Medical College). Forskolin was purchased from Enzo Life Sciences (Farmingdale, NY), and DDA (2′, 5′‐dideoxyadenosine) from CalBiotech (Spring Valley, CA).

### Western blotting

Tissue samples were processed for western blots in a manner similar to Roa et al. (2014) and Roa and Tresguerres (2016). In brief, 20 *μ*g of proteins were separated on 7.5% polyacrylamide mini gel (60 V 15 min, 200 V 45 min) and transferred to a polyvinylidene difluoride (PVDF) membrane (Bio‐Rad). After transfer, PVDF membranes were incubated in blocking buffer (tris‐buffered saline, 1% tween, 5% skim milk) at room temperature (RT) for 1 h and incubated in the primary antibody at 4°C overnight (anti‐dfsAC = 3 *μ*g/mL; anti‐NKA = 1.5 *μ*g/mL). PVDF membranes were washed 3× (10 min each) in tris‐buffered saline + 1% tween (TBS‐T), incubated in the appropriate anti‐rabbit or anti‐mouse secondary antibodies (1:10,000) at RT for 1 h, and washed 3× (10 min each) in TBS‐T. Bands were made visible through addition of ECL Prime Western Blotting Detection Reagent (GE Healthcare, Waukesha, WI) and imaged and analyzed in a BioRad Universal III Hood using ImageQuant software (BioRad). PVDF membranes incubated in blocking buffer with anti‐dfsAC antibodies and 300‐fold excess blocking peptide served as control and did not show any bands.

### Immunostaining of tissue sections and isolated nuclei

Tissue samples fixed and stored in 70% ethanol as described above were processed into 7 *μ*m histological sections following Roa et al. (2014) and Roa and Tresguerres (2016). After blocking (1 h, PBS, 2% normal goat serum, 0.02% keyhole limpet hemocyanin, pH 7.7), sections were incubated in the anti‐dfsAC primary antibodies at 4°C overnight (12 *μ*g/mL). Slides were washed 3× in PBS, incubated in goat anti‐rabbit Alexa Fluor 488 secondary antibodies (1:500) at RT for 1 h, incubated with the nuclear stain Hoechst 33342 (Invitrogen) (5 *μ*g/mL) for 5 min, washed 3× in PBS, and permanently mounted in Fluorogel with tris buffer (Electron Microscopy Sciences, Hatfield, PA). Immunofluorescence was detected using an epifluorescence microscope (Zeiss AxioObserver Z1) connected to a metal halide lamp and appropriate filters. Digital images were adjusted, for brightness and contrast, using Zeiss Axiovision software and Adobe Photoshop. Antigen retrieval was required for anti‐dfsAC, which involved incubating slides in heated (95°C) citrate unmasking buffer (10 mmol/L citric acid, 0.05% Tween20, pH 6.0) for 30 min following rehydration. For anti‐dfsAC, control sections incubated in blocking buffer with anti‐dfsAC antibodies and 300‐fold excess blocking peptide showed no visible sAC immunoreactivity. For localization of sAC in all gill cells at longer exposure times and for localization of sAC to the cell nucleus of histological sections, images were captured using structured illumination (Zeiss Apotome2).

Following isolation, nuclei were resuspended in 100 *μ*L of saline, allowed to settle on poly‐L‐lysine‐coated coverslips for 1 h, fixed for 20 min (3.2% paraformaldehyde, and 0.3% glutaraldehyde in shark saline, RT), incubated in blocking buffer for 1 h (RT), incubated in anti‐dfsAC antibodies at 4°C overnight (12 *μ*g/mL), and processed for immunocytochemistry as described above.

### Co‐expression of sAC with NKA, VHA, tmACs

For immunolocalization of sAC to NKA‐rich cells, sections were processed as described above then incubated in a mixture of anti‐dfsAC (rabbit) and anti‐NKA (mouse) antibodies overnight (4°C), followed by a mixture of goat anti‐rabbit Alexa Fluor 488 and goat anti‐mouse Alexa Fluor 568 secondary antibodies as described in Roa et al. (2014). For immunolocalization of sAC to VHA‐rich cells, sections were first incubated in anti‐dfsAC antibodies alone overnight (4°C). This was followed by incubation in goat anti‐rabbit Alexa Fluor 488 secondary antibodies (1 h, RT), blocking buffer (1 h, RT), anti‐VHA antibodies (4 h, RT), goat anti‐rabbit Alexa Fluor 555 secondary antibodies (1 h, RT), and processed as described above. Similarly, to show co‐expression of sAC and tmACs, sections were incubated alone in anti‐dfsAC antibodies overnight (4°C), followed by goat anti‐rabbit Alexa Fluor 555 secondary antibodies (1°h, RT) and BODIPY‐FL Forskolin dye (30 min, RT) as described in Roa and Tresguerres (2016) (anti‐dfsAC = 12 *μ*g/mL; anti‐VHA = 6 *μ*g/mL; anti‐NKA = 6 *μ*g/mL; BODIPY‐FL Forskolin = 10 *μ*mol/L).

### cAMP assays

Tissue homogenates and isolated nuclei were incubated for 30 min at RT on an orbital shaker (300 rpm) in 100 mmol/L Tris (pH 7.5), 5 mmol/L ATP, 10 mmol/L MgCl_2_, 0.1 mmol/L MnCl_2_, 0.5 mmol/L IBMX, 1 mmol/L dithiothreitol (DTT), 20 mmol/L creatine phosphate (CP), and 100 U·mL^−1^ creatine phosphokinase (CPK). For inhibition of sAC activity, samples were incubated in 50 *μ*mol/l KH7, a concentration known to inhibit elasmobranch sAC but not tmAC (Tresguerres et al. 2010c; Roa and Tresguerres 2016). For inhibition of tmAC activity, tissue homogenates were incubated in 10 *μ*mol/L forskolin and combinations 50 *μ*mol/L KH7 and 100 *μ*mol/L DDA, a concentration known to inhibit elasmobranch tmACs but not sAC (Roa and Tresguerres 2016). cAMP concentrations were determined using DetectX Direct Cyclic AMP Enzyme Immunoassay (Arbor Assays, Ann Arbor, MI).

### Statistical analysis

All quantitative data from experimental groups were analyzed for significant differences using a one‐way repeated measures ANOVA and Tukey's Multiple Comparison Test (*P* < 0.001). All analysis completed using GraphPad Prism software (GraphPad, La Jolla, CA).

## Results

### sAC is present in acid‐base regulatory gill cells

**Figure 1:**
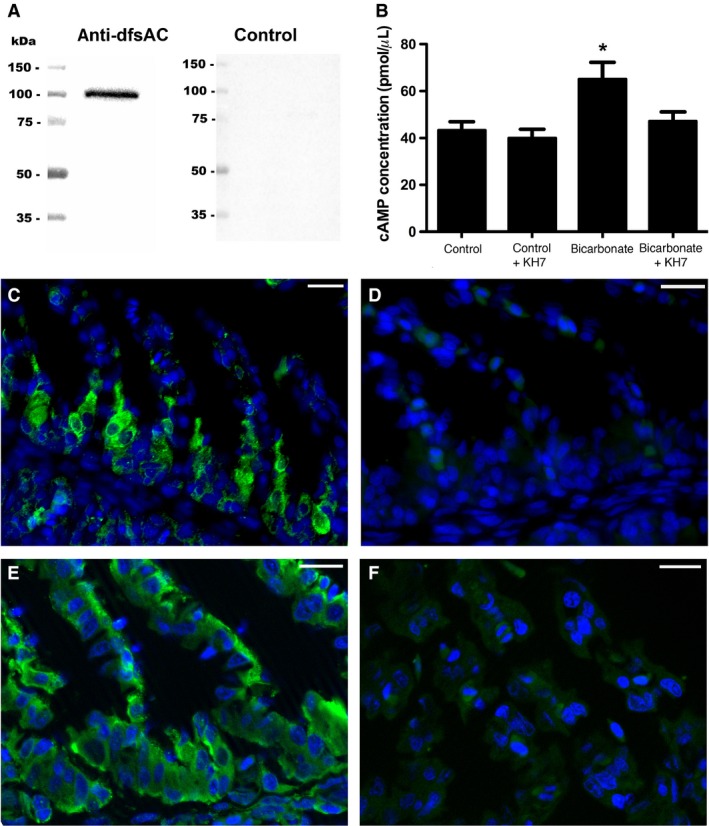
sAC in leopard shark gills. (A) anti‐dogfish sAC (dfsAC) antibodies specifically recognized the 110‐kDa shark sAC in western blots from 20 *μ*g of gill crude homogenate, but not in peptide preabsorption control blots. (B) 40 mmol/L HCO_3_^−^ (+DMSO) stimulated cAMP production in gill crude homogenates, which was blocked by the sAC‐specific inhibitor KH7 (*n* = 5, *P* < 0.001). (C–D) sAC immunoreactivity was highly abundant in acid‐base regulatory cells from leopard shark gills (C, green), but not in peptide preabsorption control sections (D). (E–F) At longer exposure times, sAC immunoreactivity was present in all leopard shark gill cells (E, green), but not in peptide preabsorption control sections (F). Nuclei stained in blue. Scale bars = 20 *μ*m. cAMP, cyclic adenosine monophosphate; sAC, soluble adenylyl cyclase.

**Figure 2:**
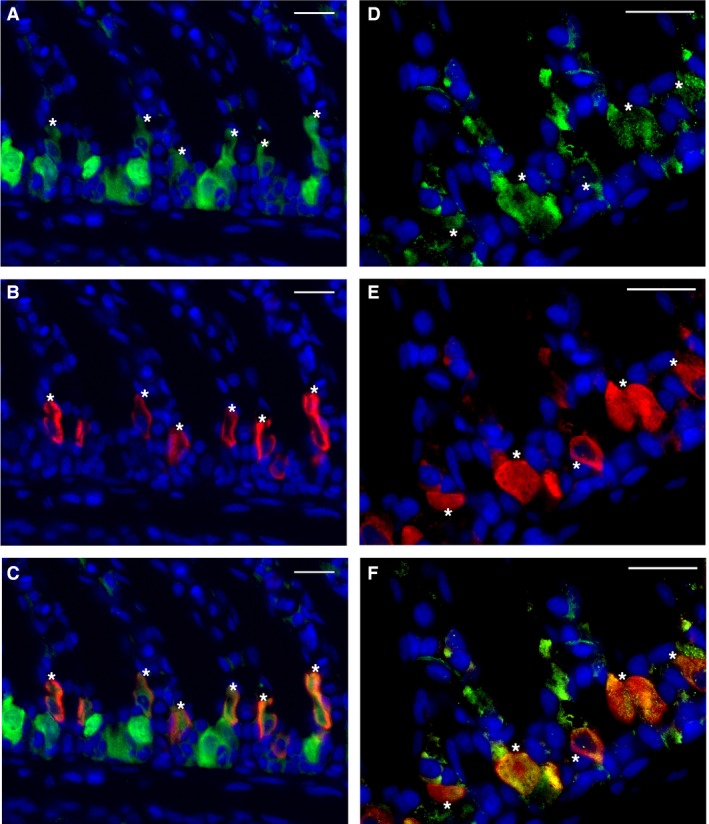
sAC in Na^+^/K^+^‐ATPase (NKA)‐rich and vacuolar‐type H^+^‐ATPase (VHA)‐rich gill cells. (A–C) sAC immunoreactivity (A, green) and NKA immunoreactivity (B, red) are co‐expressed together in cells along the interlamellar gill region (C). Cells marked with asterisks (*) co‐express sAC and NKA. (D–F): sAC immunoreactivity (D, green) and VHA immunoreactivity (E, red) colocalize together in the cytoplasm of cells along the interlamellar gill region (F). Cells marked with asterisks (*) co‐express sAC and VHA. Nuclei stained in blue. Scale bars = 20 *μ*m. NKA, Na_+_/K_+_‐ATPase; VHA, vacuolar‐type H^+^‐ATPase; sAC, soluble adenylyl cyclase.

Anti‐dfsAC antibodies recognized the predicted ~110 kDa band for shark sAC in western blots from leopard shark gill extracts, but not in control blots (Fig. 1A). Gill extracts incubated in 40 mmol/L HCO_3_^−^ produced significantly more cAMP than gill extracts incubated in control conditions (65 ± 7 pmol *μ*L^−1^ vs. 43 ± 3 pmol *μ*L^−1^, Fig. 1B); HCO_3_^−^‐stimulated cAMP production was inhibited by the sAC‐specific small molecule inhibitor KH7 (Fig. 1B), which is a hallmark of sAC activity. In histological sections, sAC immunofluorescence was present throughout the cytoplasm of acid‐base regulatory cells along the interlamellar gill region (Fig. 1C) but was absent in control sections (Fig. 1D). Increasing the exposure time revealed sAC is also present in all other cell types (Fig. 1E), although at much lower abundance. However, this sAC immunostaining was also specific as no signal was present in time‐matched control sections, also imaged at longer exposure times (Fig. 1F). Additionally, double immunolabeling demonstrated sAC is highly abundant in both shark acid‐base regulatory cell types, as strong sAC immunoreactivity was co‐expressed with either NKA or VHA in acid‐secreting NKA‐rich cells (Fig. 2A–C) and base‐secreting VHA‐rich cells (Fig. 2D–F).

### sAC and tmACs in leopard shark gills

**Figure 3:**
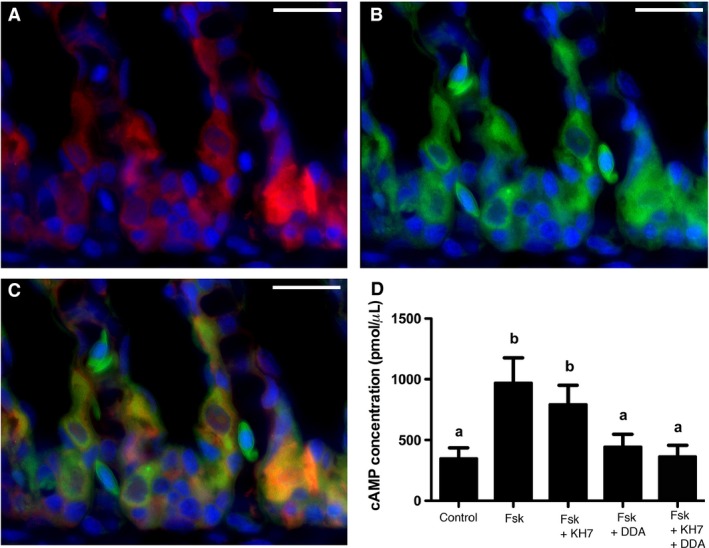
sAC and transmembrane adenylyl cyclases (tmACs) in leopard shark gills. (A–C) sAC immunoreactivity (A, red) and BODIPY‐FL forskolin‐labeled tmACs (B, green) are found together in cells along the interlamellar gill region (C). (D) Forskolin stimulated cAMP production in gill crude homogenates, which was blocked by the tmAC‐specific inhibitor 2′, 5′‐dideoxyadenosine (DDA) but not by the sAC‐specific inhibitor KH7 (*n* = 4, *P* < 0.001). Nuclei stained in blue. Scale bars = 20 *μ*m. cAMP, cyclic adenosine monophosphate; sAC, soluble adenylyl cyclase.

Similar to reports from round ray (Roa and Tresguerres 2016), tmACs were present and co‐expressed with sAC in leopard shark gill acid‐base regulatory cells, indicating the presence of two separate sources of cAMP in these cells (Fig. 3A–C). Gill extracts incubated in the tmAC agonist forskolin produced significantly more cAMP than gill extracts incubated in control conditions (971 ± 206 pmol *μ*L^−1^ vs. 348 ± 90 pmol *μ*L^−1^, Fig. 3D). Forskolin‐stimulated cAMP production was inhibited by the tmAC‐specific inhibitor DDA but not inhibited by the sAC‐specific inhibitor KH7.

### sAC is present throughout leopard shark tissues

**Figure 4:**
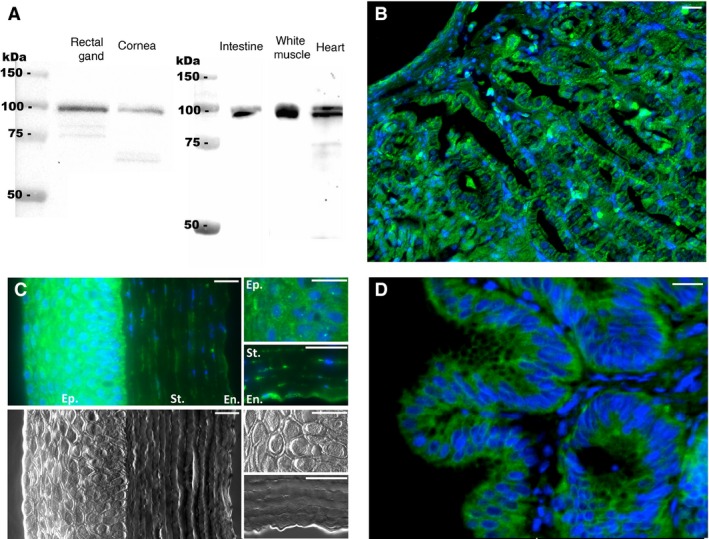
sAC in leopard shark tissues. (A) anti‐dogfish sAC (dfsAC) antibodies specifically recognized the 110‐kDa shark sAC in western blots using crude homogenates of rectal gland, cornea, intestine, white muscle, and heart (20 *μ*g total protein in each lane). (B–D) sAC immunoreactivity (green) was highly abundant in leopard shark (B) rectal gland, (C) cornea – sAC immunoreactivity on top 3 panels, corresponding DIC images on lower 3 panels – with sAC immunoreactivity strongest in the epithelium (Ep.) and stroma (St.) but also present in endothelial cells (En.), and (D) intestine. Nuclei stained in blue. Scale bars = 20 *μ*m. sAC, soluble adenylyl cyclase.

Anti‐dfsAC antibodies recognized the predicted ~110 kDa band for shark sAC in western blots from leopard shark rectal gland, cornea, intestine, white muscle, and heart (Fig. 4A). The smaller molecular weight bands showed in Figure 4A likely correspond to sAC splice variants or isoforms as described for mammalian sAC (Geng et al. 2005; Farrell et al. 2008); however, these bands are much fainter compared to the 110 KDa band and only appear at relatively long exposure time.

In histological sections, sAC immunofluorescence was present throughout the cytoplasm of cells in the rectal gland, eye, and intestine. In the rectal gland, sAC immunofluorescence was strongest in the tubule cells, which secrete Na^+^ and Cl^−^ ions into the gland lumen (Fig. 4B). In the cornea, sAC immunofluorescence was strongest in epithelial cells and stroma keratocytes, and it was also present in endothelial cells (Fig. 4C), and in the intestine sAC was highly abundant in all epithelial cells (Fig. 4D). In addition, some cells showed sAC immunostaining in the nucleus, which was further explored in gill and rectal gland tissues as described below. sAC immunofluorescence was absent in control sections incubated in anti‐dfsAC antibodies and 300‐fold excess sAC peptide.

### sAC is present and active in leopard shark cell nuclei

**Figure 5:**
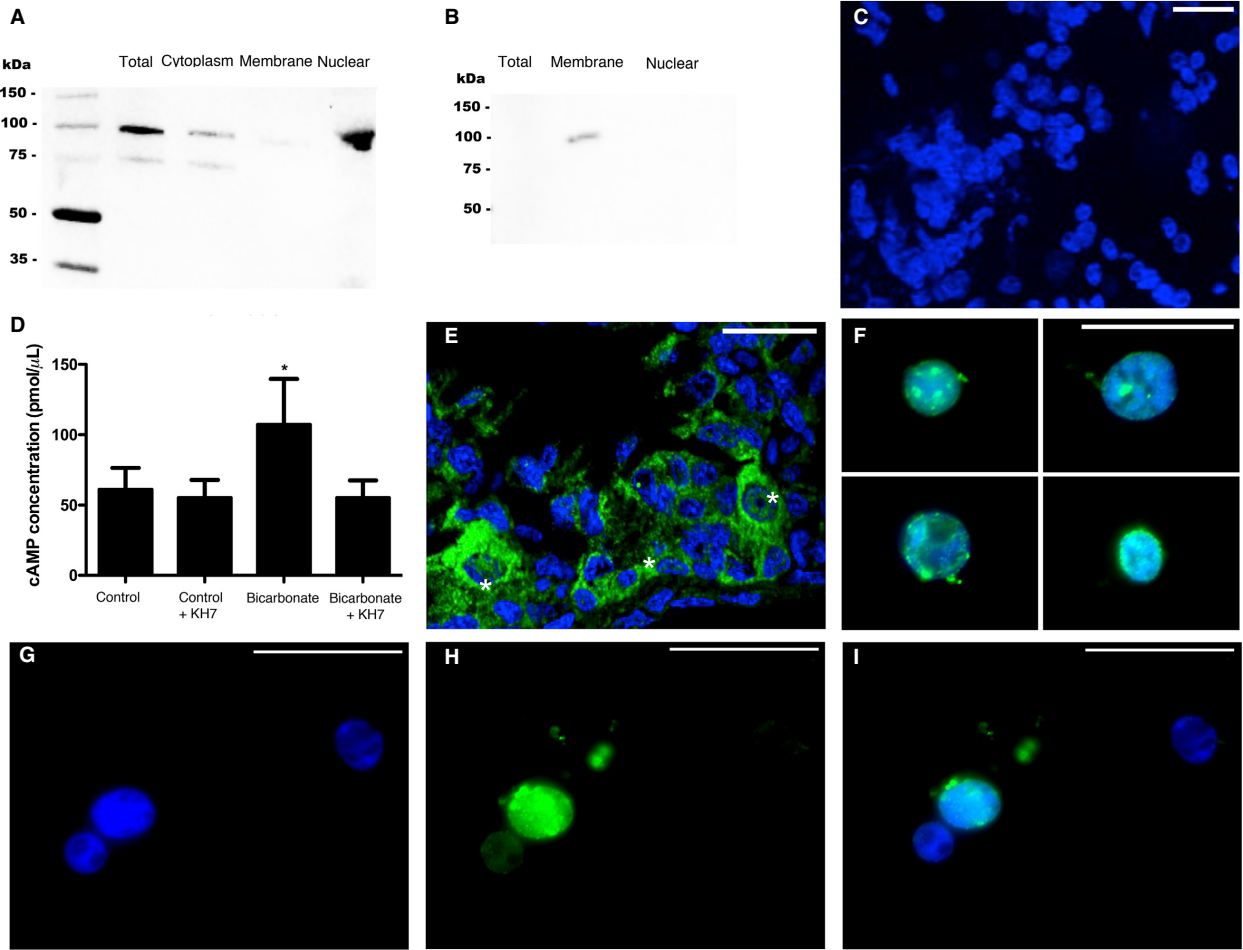
sAC in nuclei of leopard shark gill cells. (A) anti‐dogfish sAC (dfsAC) antibodies specifically recognized the 110‐kDa shark sAC in western blots from 20 *μ*g of gill total crude homogenate, cytoplasmic, and nuclear cell fractions; but not in the membrane fraction. (B) anti‐Na^+^/K^+^‐ATPase (NKA) antibodies specifically recognized the 100‐kDa NKA band in the membrane fraction, but not in total crude homogenate or in the nuclear fraction. (C) Representative image of Hoechst‐stained isolated nuclei (blue) gathered using nuclear enrichment protocol. (D) 40 mmol/L HCO_3_^−^ (+DMSO) stimulated cAMP production in nuclei isolated from gill cells, which was blocked by the sAC‐specific inhibitor KH7 (*n* = 5, *P* < 0.001). (E–F) sAC immunoreactivity (green) was highly abundant in some nuclei in gill histological sections (E, *) – imaged using epifluorescence structured illumination – and in nuclei isolated from gill cells (F). Nuclei also stained in blue. (G–I) Representative images of isolated nuclei (G, blue) and sAC immunoreactivity (H, green), showing sAC is present in some, but not all, nuclei isolated from leopard shark gill cells (I). Scale bars = 20 *μ*m. cAMP, cyclic adenosine monophosphate; sAC, soluble adenylyl cyclase.

Differential centrifugation separated crude homogenate, cytoplasm, membrane, and nuclear fractions from gill extracts. Anti‐dfsAC antibodies recognized the ~110 kDa band for shark sAC in western blots from gill crude homogenate, cytoplasm, and nuclear fractions, but no band was detected in the membrane fraction, consistent with the lack of transmembrane domains in the sAC protein, and also suggesting sAC in shark gill cells is not strongly associated with membrane proteins (Fig. 5A). Unfortunately, commercial antibodies against the mammalian nuclear marker proteins CREB and histone did not work in shark tissues. Thus, validation of the cell fractionation protocol was performed by western blots that detected Na^+^/K^+^‐ATPase in the membrane fraction only (Fig. 5B), and by visual inspection that confirmed enrichment of Hoechst‐stained nuclei in the nuclear fraction (Fig. 5C).

**Figure 6:**
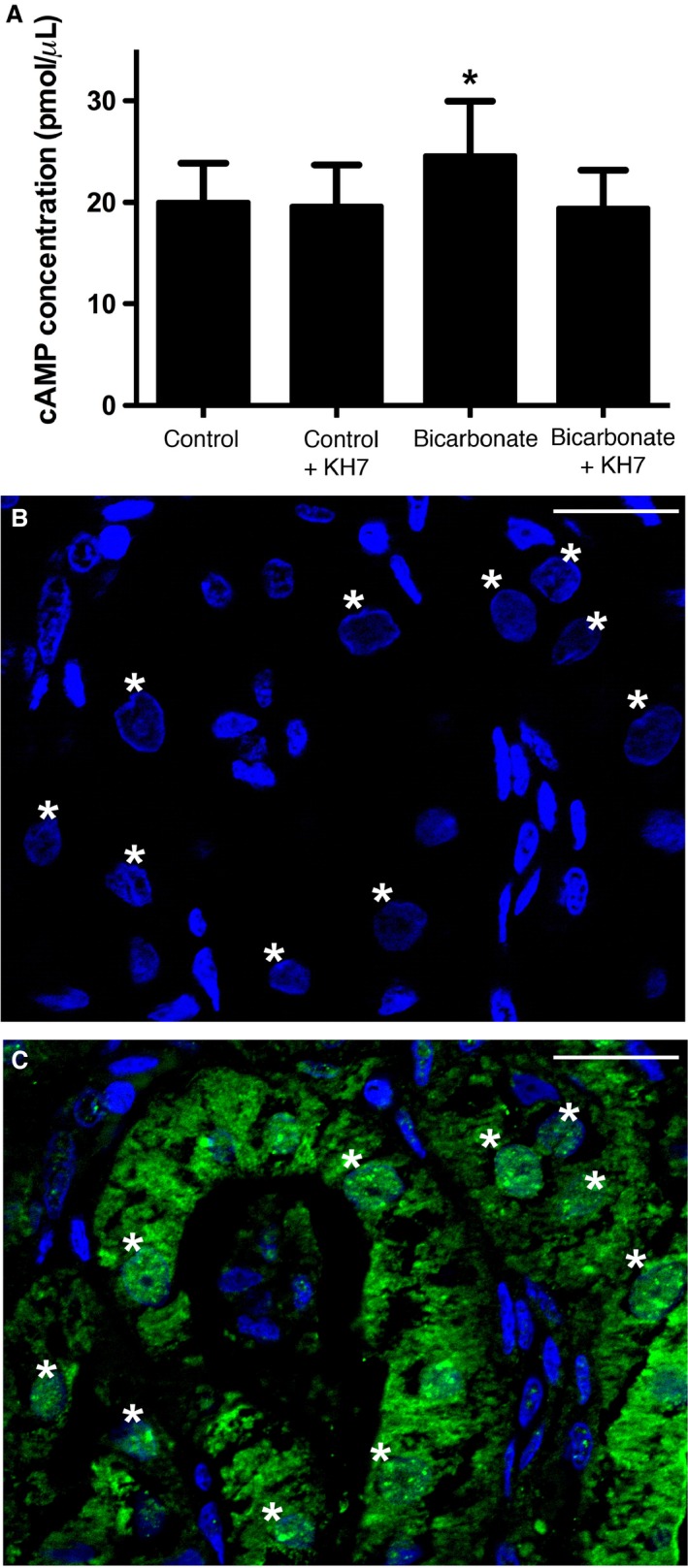
sAC in nuclei of leopard shark rectal gland cells. (A) 40 mmol/L HCO_3_^−^ (+DMSO) stimulated cAMP production in nuclei isolated from rectal gland cells, which was blocked by the sAC‐specific inhibitor KH7 (*n* = 5, *P* < 0.001). (B–C) nuclei (B, C, blue) and sAC (C, green) colocalize in rectal gland cells. Images acquired using epifluorescence structured illumination and sAC‐positive nuclei marked with asterisks (*). Scale bars = 20 *μ*m. cAMP, cyclic adenosine monophosphate; sAC, soluble adenylyl cyclase.

The presence of sAC in gill cell nuclei was additionally confirmed by cAMP activity assays on isolated nuclei, and by immunostaining of gill sections and isolated nuclei. Isolated gill nuclei incubated in 40 mmol/L HCO_3_^−^ produced significantly more cAMP than nuclei incubated in control conditions (107 ± 32 pmol *μ*L^−1^ vs. 61 ± 15 pmol *μ*L^−1^, Fig. 5D), and this HCO_3_^−^‐stimulated activity was inhibited by the sAC‐specific small molecule inhibitor KH7 (Fig. 5D). sAC immunofluorescence was detected in and around nuclei from gill histological sections (Fig. 5E), as well as in isolated nuclei (Fig. 5F). However, similar to nuclei in histological sections (Figs. 3C and 5E) not all isolated gill nuclei display sAC immunofluorescence (Fig. 5G–I). Similar to gill, rectal gland cell nuclei display HCO_3_^−^‐stimulated, KH7‐sensitive cAMP producing activity, and sAC immunofluorescence was detected in and around some, but not all, nuclei from rectal gland histological sections (Fig. 6).

## Discussion

In this study, we investigated the cellular and subcellular localization of the evolutionarily conserved acid‐base sensor sAC in various leopard shark tissues. We found sAC highly abundant throughout the cytoplasm of gill NKA‐ and VHA‐rich cells, which respectively are acid‐ and base‐secreting cells responsible for blood acid‐base regulation. Additionally, sAC was co‐expressed with tmACs in these cells, an indication that leopard sharks have two sources of cAMP in gill acid‐base regulatory cells. sAC was also present in other tissues such as rectal gland, eye, intestine, white muscle, and heart, where it likely regulates tissue‐specific responses to acid‐base conditions. Finally, sAC was found associated with the cell nucleus of certain cells, where we propose it may regulate gene expression.

The presence of sAC in gill VHA‐rich base‐secreting cells has been previously reported in dogfish shark (Tresguerres et al. 2010c) and round ray (Roa and Tresguerres 2016). Extensive research on those two species has elucidated a mechanism for sensing and counteracting of blood alkalosis, whereby sAC in base‐secreting cells senses blood alkalosis and triggers the translocation of VHA from cytoplasmic vesicles to the basolateral membrane. From that location, VHA moves H^+^ into the blood and energizes excretion of HCO_3_^−^ into seawater via the apical anion exchanger pendrin (Tresguerres et al. 2005,2007,2010b; Roa et al. 2014; Roa and Tresguerres 2016) reviewed in Tresguerres 2016). Thus, our evidence would suggest that the sAC‐dependent mechanism to sense and counteract blood alkalosis applies to gills from all marine elasmobranchs.

In addition to base‐secreting VHA‐rich cells, sAC was highly abundant in NKA‐rich acid‐secreting cells. We propose that sAC in these cells acts to downregulate acid secretion during blood alkalosis, but experimental evidence would be required to support this model. Another interesting topic for future research is the interaction between sAC and tmACs, as these two cAMP‐producing enzymes were co‐expressed in leopard shark gill epithelial cells. In agreement with the cAMP microdomain model that predicts multiple cAMP sources with different downstream effects (reviewed in Cooper 2003; Tresguerres et al. 2011), sAC and tmACs have been reported to exert opposing effects on the VHA translocation in round ray base‐secreting cells (Roa and Tresguerres 2016), as well as having differential effects on fish intestinal ion and water transport (Tresguerres et al. 2010b; Carvalho et al. 2012) and hagfish heart beat rate (Wilson et al. 2016). The co‐expression of sAC and tmAC in both acid‐ and base‐secreting cells found in this study suggests a more extensive and complex regulation of acid‐base transport by cAMP in the shark gill epithelium.

In this study, sAC was found more highly abundant in acid‐base regulatory cells of leopard shark, but sAC was more evenly expressed in all gill epithelial cells of round ray in a previous study (Roa and Tresguerres 2016). However, increasing the exposure time during immunofluorescence detection revealed sAC is also present in all other gill epithelial cells of leopard shark, only at lower abundance. sAC localization in acid‐ and base‐secreting cells was not studied in detail in dogfish, although strong presence in some pillar cells was noted (Tresguerres et al. 2010c). In any case, sAC is likely to play additional physiological roles in non‐acid‐base regulatory gill cells.

Elasmobranch fishes regularly experience pronounced blood acid‐base disturbances as a result of their normal activities and ecophysiology. For example, they might experience metabolic acidosis as a result of exhaustive exercise (Richards et al. 2003), metabolic alkalosis in the post‐feeding period (Wood et al. 2005, 2007a, 2010), and elevations in plasma HCO_3_^−^ as a result of environmental oxygen, CO_2_, and temperature levels (reviewed in Heisler 1988). Our finding that sAC protein is expressed in multiple shark tissues in addition to the gills, namely rectal gland, eye, intestine, white muscle, and heart, suggests sAC plays multiple general and organ‐specific roles in these tissues. Furthermore, sAC has also been reported in dogfish shark red blood cells and testis (Tresguerres et al. 2014).

Based on published effects of HCO_3_^−^ and cAMP, and reports from mammalian sAC, we hypothesize sAC may have multiple physiological functions in the shark tissues examined. In the rectal gland, sAC may regulate NaCl secretion, as this function is dependent on the blood acid‐base status (Wood et al. 2007b), and it is well established to be regulated by cAMP as originally reported by (Stoff et al. (1977) and confirmed multiple times since. Additionally, alkalosis induces important transcriptional and enzymatic changes in rectal gland (Walsh et al. 2006; Deck et al. 2013), which may be mediated by sAC located in the nucleus as discussed below.

In the cornea, sAC is likely activated by high HCO_3_^−^ in the aqueous humor (Maren et al. 1975) where it can facilitate fluid and ion balance necessary for proper vision. The cornea of elasmobranchs is an interesting tissue because it does not swell when placed in diluted saline solutions or even distilled water (Smelser 1962; Candia et al. 1976), unlike the mammalian cornea where swelling occurs and can lead to an opaque cornea, blurred vision, and even blindness. However, similar to sharks (this study), sAC is expressed throughout the mammalian cornea (Lee et al. 2014), where it regulates chloride permeability (Sun et al. 2004) and mediates protective HCO_3_^−^‐dependent effects on cell health (Li et al. 2011). Future research could explore whether sAC in shark cornea modulates ion and fluid transport as well as cell survival and protection, and whether these potential mechanisms are relevant for human cornea health.

The potential roles of sAC in muscle are varied, as myocytes generate large amounts of CO_2_ and H^+^ (Hochachka and Somero 2002). Furthermore, sAC has been recently shown to modulate heart beat rate in hagfish in response to HCO_3_^−^ (Wilson et al. 2016). The presence of sAC in shark heart raises the question of whether the mechanism reported for hagfish also applies to elasmobranchs and other fishes.

Finally, sAC was abundantly expressed in shark intestinal epithelial cells, which resembles the intestine of marine teleost fish (Tresguerres et al. 2010b). However, while sAC in the marine bony fish intestine modulates NaCl and water absorption for hypo‐osmoregulation (Tresguerres et al. 2010b; Carvalho et al. 2012), marine sharks are osmoconfomers or slight hyper‐regulators and therefore sAC in the shark intestine must have some other function(s). Two testable hypotheses include the potential sAC‐dependent regulation of intestinal nutrient and ammonia absorption, both of which are upregulated during the postfeeding alkaline tide (Wood and Bucking 2011). However, it is important to stress the potential sAC‐dependent functions in shark rectal gland, cornea, muscle, heart and intestine mentioned above are speculative and their confirmation awaits experimental evidence.

Due to low availability of custom‐made anti‐shark sAC antibodies, we only screened for the presence of sAC protein in tissues that (Aronson et al. 1982) had not been previously examined in elasmobranchs, and (Baudouin‐Legros et al. 2008) we are planning to conduct follow‐up studies on. However, similar to humans (Uhlén et al. 2015), www.proteinatlas.org), sAC is likely to be ubiquitously expressed throughout elasmobranch tissues. This opens exciting research opportunities to elucidate sAC universal and cell‐specific physiological roles.

In addition to the expected cytosolic localization, sAC was present in or around the nucleus of cells of the shark tissues examined, especially in gill, cornea, and rectal gland. We further investigated this potential subcellular localization of sAC by western blotting, enzyme activity assays, and immunolabeling in isolated nuclei from gill cells. Western blotting confirmed the presence of the predicted 110 KDa band corresponding to elasmobranch sAC (37, 54, this study). And enzyme activity assays confirmed HCO_3_^−^‐stimulated and KH7‐sensitive cAMP production, a hallmark of sAC activity (Hess et al. 2005). However, it is important to note that although KH7 significantly reduced sAC activity in gill and rectal gland nuclei, we did observe KH7 insensitive cAMP in our preparations, which could be explained by contamination with sAC or tmACs from whole cells or other cellular components, as well as from tmACs presence around the nucleus. To rule these possibilities out, we first verified that the presumed nuclear‐enriched fraction did not contain NKA, which would have been an indication of contamination with cell plasma membrane (Li and Donowitz 2014). Secondly, visual inspection of samples incubated with the DNA stain Hoechst 33342 confirmed the presumed nuclear‐enriched fraction was indeed enriched in nuclei and devoid of whole cells. Finally, immunolabeling of isolated cell nuclei confirmed the presence of sAC in and around nuclei. Unfortunately, the tools (i.e., tmAC‐specific antibodies) necessary to explore tmAC localization in or around the nucleus have yet to be developed for shark tmACs, and this remains an active area of investigation. Altogether, the evidence demonstrates sAC is present in the nuclei of shark gill cells and based on a subset of experiments, sAC is also present in other shark tissues, including rectal gland, cornea, and intestine. Interestingly, sAC was not present in all nuclei, suggesting sAC might move to the cell nucleus in response to acid‐base stress (e.g., alkalosis) in order to regulate nuclear functions.

Nuclear sAC localization has previously only been described in cultured mammalian cell lines (Zippin et al. 2003, 2004; Schmitz et al. 2014), in cancer cells (Desman et al. 2014; Zippin et al. 2010), and in liver cells (Zippin et al. 2004). To the best of our knowledge, sAC in the nucleus of liver cells is the only other report (aside from this study) of nuclear sAC in a nonpathological, native organ, and similar to our results in sharks, only a fraction of cells had nuclear sAC immunostaining (Zippin et al. 2004). In the nucleus of mammalian cells, the only proposed role of sAC is to regulate the expression of certain genes *via* phosphorylation of the transcription factor CREB (Zippin et al. 2004; Baudouin‐Legros et al. 2008; Li et al. 2011; Schmitz et al. 2014). Exploring this model in shark cells awaits the development of antibodies that specifically recognize shark CREB in its unphosphorylated and phosphorylated states.

## Perspectives and Significance

Although sAC was already known to be present in elasmobranch gills and to sense and regulate blood acid‐base status (Tresguerres et al. 2010c; Roa and Tresguerres 2016), its presence in organs not specialized in systemic acid‐base regulation indicate multiple other physiological functions. The presence of sAC in the nucleus of shark cells opens new avenues of research on regulation of gene expression in response to acid‐base disturbances. Furthermore, since sAC is an evolutionarily conserved acid‐base sensor (Buck et al. 1999; Chen et al. 2000), nuclear sAC might serve a similar role in many other organisms. Elasmobranch fishes regularly experience large variations in pH, CO_2_, and HCO_3_^−^ levels as part of their normal physiology, and elasmobranch sAC is fairly well characterized at the biochemical, pharmacological, and cellular level. Thus, elasmobranch fishes are an excellent model to study acid‐base sensing by sAC and its multiple downstream physiological effects. Another important finding of our study is that shark cells, like mammalian cells, have at least two different sources of cAMP and therefore must also possess cAMP signaling microdomains. This novel information for future research on cAMP signaling, as well for revisiting studies on cAMP that were published before the existence of sAC (and microdomains) was known.

## Conflict of Interest

All the authors declared no competing interests.
